# Supplementary material for: Coverage of climate change in introductory biology textbooks, 1970–2019
Source: PLoS One. 2022 Dec 21;17(12):e0278532. doi: 10.1371/journal.pone.0278532 (PMC9770425; doi:10.1371/journal.pone.0278532)
Supplement: S1 Appendix — (DOCX) [file pone.0278532.s001.docx]

Appendix A: List of textbooks used

Decade: 1970s

1971: The Study of Biology (2e/4) by Baker, Allen

1972: Biology Today (1e/3) by CRM Books

1972: Biological Science (2e/6) by Keeton

1972: Invitation to Biology (1e/5) by Curtis

1973: Life on Earth (1e/1) by Wilson et al

1973: Elements of Biology (1e/3) by Levy

1977: Biology (1e/1) by Herreid

1979: Biology (1e/4) by Arms, Camps

1979: Biology (3e/5) by Curtis

Decade: 1980s

1981: Invitation to Biology (3e) by Curtis, Barnes

1981: Biology: The Science of Life (1e) by Wallace, King, Sanders

1982: Biology (2e) by Arms, Camp

1983: Elements of Biological Science (3e) by Keeton, McFadden

1983: Biology (4e) by Curtis

1983: Life: The Science of Biology (1e) by Purves, Orians

1984: Biology: Unity & Diversity of Life (3e) by Starr, Taggart

1985: Biology (2e) by Villee, Solomon, Davis

1985: Invitation to Biology (4e) by Curtis, Barns

1989: Biology: Unity & Diversity (5e) by Starr, Taggart

1989: The Nature of Life (1e) by Postlethwait, Hopson

1989: Biology (5e) by Curtis, Barnes

Decade: 1990s

1990: Biology (3e) by Mader

1992: Biology: The World of Life (6e) by R. Wallace

1992: The Nature of Life (2e) by Postlethwait, Hopson

1992: Biology (3e) by Raven, Johnson

1993: Biology: Unity & Diversity (3e) by Campbell

1993: Biology, Life on Earth (4e) by Audesirk

1993: Biological Science (5e) by Keeton, Gould

1996: Biology (4e) by Solomon, Berg, Martin, Villiee

1996: Biology: Understanding Life (2e) by Alters

1998: Life (3e) by R. Lewis

1999: Biology by (5e) by Solomon, Berg, Martin

1999: Biology: Life on Earth (5e) by Audesirk, Audesirk

Decade: 2000s

2002: Biology (6e) by Raven, Johnson

2002: Biology (6e) by Campbell, Reece, Taylor

2002: Biology (6e) by Solomon, Berg, Martin

2004: Biology, Unity & Diversity of Life (10e) by Starr, Taggart

2004: Life: The Science of Biology (7e) by Purves, Sadava, Orians, Heller

2005: Biology (7e) by Campbell, Reece

2005: Biology: Life on Earth (7e) by Audesirk T, Audesirk G, Byers BE

2006: Biology (1e) by Alters, Alters

2006: The Living World (4e) by G. Johnson

2006: Biology Concepts & Connections (5e) by Campbell, Reece

2008: Inquiry into Life (12e) by Mader

2008: Biology (1e) by Brooker, Widmaier, et al

2008: Life: The Science of Biology (8e) by Sadava, Heller, et al

2008: Biology: Life on Earth (with physiology) (8e) by Audesirk, Audesirk, Byers

2009: Biology: Concepts & Investigations (1e) by Hoefnagels

Decade: 2010s

2010: Essentials of the Living World (3e) by Johnson G

2011: Biology (9e) by Raven, Johnson et al

2014: Concepts of Biology (3e) by Mader

2014: Biology (10e) by Mason, Losos, Singer

2015: Biology: Concepts & Connections (8e) by Reece et al

2015: Biology: Concepts & Investigations (3e) by Hoefnagels

2017: Biology (11e) by Raven, Johnson et al

2017: Life: The Science of Biology (11e) by Sadava, Heller, et al

2018: What is Life? (4e) by Phelan
