# Supplementary material for: Coverage of climate change in introductory biology textbooks, 1970–2019
Source: PLoS One. 2022 Dec 21;17(12):e0278532. doi: 10.1371/journal.pone.0278532 (PMC9770425; doi:10.1371/journal.pone.0278532)
Supplement: Attachment — Submitted filename: ResponseToReviewers.docx [file pone.0278532.s002.docx]

September 22, 2022

Re: Manuscript revisions for “Coverage of climate change in introductory biology textbooks, 1970-2019” [PONE-D-22-22659]

Dear Dr. Gomez and esteemed reviewers,

Thank you for the time you spent reviewing our paper, and for the thoughtful and helpful comments on our manuscript. We hope that our revisions reflect our appreciation for your expert review.

Reviewer 1

We greatly appreciate the suggestion to **introduce the topic of study earlier in the introduction**. We have included a paragraph at the start of the introduction (lines 39-43) to clarify the focus and address the organization of that section.

We have **refined each of the four hypotheses to clarify connections** between the “textbooks as historical documents” section and our predictions (lines 123-146).

Also, we have corrected a setting to make **the dataset publicly available**. Please accept our apologies for the error and our thanks for pointing out this problem. You should be able access the data at: <https://doi.org/10.5061/dryad.tht76hf2f>

We humbly offer our thanks for such positive and constructive notes about our paper.

Reviewer 2

Thank you for the suggestions to elaborate on our argument and broaden our conclusions for an international audience. We have made the following changes and believe they have greatly improved the paper.

To improve the conceptualization of our argument, we **added two paragraphs attempting to address curricular theory and decision-making and its relevance to our argument prior to our predictions** (lines 112-120). Thank you for guiding us to the Goodlad book and associated curriculum theories.

We wholeheartedly agree with the premise that generalizability is warranted. We **added two paragraphs under an additional header (“International Impacts”) in the conclusions section** (lines 441-451).

Lastly, we agree that the topic of climate change is more broadly addressed in the educational system. As such, a full analysis of the topic would need to be much deeper and more complex than we have presented. However, we were concerned about addressing a wider educational context involving other subjects since our sample of textbooks was limited to introductory biology. Therefore, we have added an **acknowledgement of the limited nature of our study** (lines 463-466). We hope that this acknowledgement is sufficient to address your justified concern. Thank you for your assistance and guidance in developing a more nuanced paper.
